# Hepatic Autonomic Nervous System and Neurotrophic Factors Regulate the Pathogenesis and Progression of Non-alcoholic Fatty Liver Disease

**DOI:** 10.3389/fmed.2020.00062

**Published:** 2020-02-27

**Authors:** Muhammad Amir, Michael Yu, Peijian He, Shanthi Srinivasan

**Affiliations:** ^1^Division of Digestive Diseases, Department of Medicine, Emory University School of Medicine, Atlanta, GA, United States; ^2^Department of Medicine, Emory University School of Medicine, Atlanta, GA, United States; ^3^Research-Gastroenterology, Atlanta VA Health Care System, Decatur, GA, United States

**Keywords:** autonomic nervous system, fibrosis, GDNF, NAFLD, NASH, neurotrophic factors

## Abstract

Non-alcoholic fatty liver disease represents a continuum of excessive hepatic steatosis, inflammation and fibrosis. It is a growing epidemic in the United States of America and worldwide. Progression of non-alcoholic fatty liver disease can lead to morbidity and mortality due to complications such as cirrhosis or hepatocellular carcinoma. Pathogenesis of non-alcoholic fatty liver disease is centered on increased hepatic lipogenesis and decreased hepatic lipolysis in the setting of hepatic and systemic insulin resistance. Adipose tissue and hepatic inflammation can further perpetuate the severity of illness. Currently there are no approved therapies for non-alcoholic fatty liver disease. Most of the drugs being explored for non-alcoholic fatty liver disease focus on classical pathogenic pathways surrounding hepatic lipid accumulation, inflammation or fibrosis. Studies have demonstrated that the autonomic nervous system innervating the liver plays a crucial role in regulation of hepatic lipid homeostasis, inflammation and fibrosis. Additionally, there is growing evidence that neurotrophic factors can modulate all stages of non-alcoholic fatty liver disease. Both the autonomic nervous system and neurotrophic factors are altered in patients and murine models of non-alcoholic fatty liver disease. In this review we focus on the pathophysiological role of the autonomic nervous system and neurotrophic factors that could be potential targets for novel therapeutic approaches to treat non-alcoholic fatty liver disease.

## Introduction

Non-alcoholic fatty liver disease (NAFLD) is rapidly becoming a significant global burden by affecting over 25% of the adult population (1). NAFLD encompasses a continuous range of liver conditions covering steatosis, steatohepatitis, fibrosis, and cirrhosis (2). It is projected to be the leading indication for liver transplantation in the next 20 years (3). The pathogenesis of NAFLD remains poorly understood and therapeutic approaches are still being explored. Currently there is no approved drug therapy for NAFLD by the Food and Drug Administration. Mechanisms for hepatic steatosis involve insulin resistance, an excess influx of fatty acids into the liver, enhanced *de novo* lipogenesis, impaired lipophagy and resultant triglyceride accumulation in hepatocytes (2, 4). Steatosis progresses to non-alcoholic steatohepatitis (NASH) when inflammation develops due to mitochondrial dysfunction, endoplasmic reticulum stress, activation of the innate immune system, and dysregulation of autophagy (2, 3). Fibrosis is primarily mediated by hepatic stellate cells (HSCs), which are mesenchymal cells and part of the repair system of injured liver. Stellate cells are quiescent in homeostatic condition, but are transformed to myofibroblast-like cells in response to oxidative stress, lipopolysaccharide (LPS), apoptotic bodies and paracrine stimuli from neighboring cells. Activated stellate cells are the main source of extracellular fibrous matrix in the development of fibrosis and cirrhosis (5–7).

Both murine and primate livers are innervated by autonomic nervous system (ANS). Sympathetic and parasympathetic branches of ANS play critical roles in energy homeostasis, liver injury and repair (8–10). Both parenchymal and non-parenchymal cells in liver express receptors for common neurotransmitters (11–14). HSCs are considered as resident hepatic neuroglia functioning as neuroendocrine cells in close proximity with hepatic nervous system. HSCs express various neuroglial marker proteins such as nestin, neural cell adhesion molecule, glial acidic fibrillary protein, and synaptophysin. HSCs also express receptors for serotonin, adrenergic or muscarinic neurotransmitters, cannabinoids and opioids (15). Despite the rich hepatic supply by ANS and its interactions with hepatic cells, its role in the pathogenesis and progression of NAFLD remains elusive.

Neurotrophic factors play an integral role in the development and function of nervous system, and are involved in pathogenesis of neurodegenerative and psychiatric disorders (16). Consequently, various neurotrophic factors have been explored as therapeutic agents in treatment of neurological disorders (17, 18). Several neurotrophic factors and their receptors are expressed in various hepatic cell types (19–21), and regulate insulin sensitivity, lipid homeostasis, cellular injury and fibrosis of the liver (22–26). However, their potential in management of liver diseases including NAFLD remains mostly unexplored. This review summarizes current knowledge about the role of ANS and neurotrophic factors in modulation of hepatic steatosis, NASH, and NASH-associated hepatic fibrosis.

## Hepatic Nervous System

The liver is regulated by both sympathetic and parasympathetic branches of the central nervous system using aminergic, peptinergic, adrenergic, and cholinergic nerve endings (8). Sympathetic nerve fibers supply the liver via the splanchnic nerve and start in celiac and superior mesenteric ganglia that are under influence of intermediolateral column of spinal cord (8, 9). In rodents, sympathetic nerves have been detected only up to the portal tracts surrounding hepatic artery and portal veins (27). It is postulated that connecting gap junctions further transmit electrical signals between the hepatocytes in rodents (28) as well as in humans (8, 9). Sympathetic nerve fibers directly supply nerve endings to hepatic lobules and along hepatic sinusoids in humans and guinea-pigs. Sympathetic nerve fibers however are absent in regenerating nodules in livers affected by cirrhosis (27, 29). The Vagus nerve supplies parasympathetic neurons originating in dorsal motor nucleus and relays their nerve endings directly to the liver or indirectly via hepatic hilar ganglia (8, 9, 30). Like sympathetic nerve fibers, parasympathetic or cholinergic fibers penetrate deep into hepatic parenchyma (31). Cholinergic fibers directly interact with HSCs and the quantity of these nerve fibers increases in fibrous septa in livers of carbon tetrachloride (CCl_4_)-treated rats (32, 33). Alpha/beta-adrenergic receptors and muscarinic cholinergic receptors are expressed by hepatocytes and HSCs (11, 12, 34–37), while nicotinic acetylcholine receptors are present in hepatocytes, Kupffer cells, macrophages, and dendritic cells (13, 14). Overall, sympathetic and parasympathetic nerve endings can relay signals to liver cells through three different mechanisms: (1) direct innervation onto or near cells by secreting norepinephrine, acetylcholine and neuropeptides such as galanin, neuropeptide Y, etc.; (2) spreading of ions or small molecules using gap junctions; (3) innervation of sinusoidal endothelial and Kupffer cells which communicate with hepatocytes via eicosanoids, cytokines, endothelin, and nitric oxide (38).

Afferent fibers of the sympathetic nervous system (SNS) and parasympathetic nervous system (PSNS) sense levels of ions, glucose, free fatty acids, cytokines as well as hormones such as glucagon like peptide-1 and cholecystokinin. This sensory information is then relayed to the hypothalamus, which in turn modulates sympathetic or parasympathetic outflow to control hepatic energy homeostasis (8, 9). Activation of SNS leads to increased gluconeogenesis and decreased glycogenesis, whereas parasympathetic activation plays an opposite role. In addition, hepatic sympathetic supply also regulates lipoprotein secretion, beta oxidation, ketone body synthesis and release (30).

### Sympathetic Nervous System and Non-alcoholic Steatohepatitis

Limited investigations have been done to study the role of SNS in pathogenesis of NASH. Overexpression of adrenergic β-receptors in cultured hepatocytes from rats or mice causes an increase in lipid accumulation (39). Hurr et al. showed that sympathetic nerve activity doubled after 10 weeks of high-fat diet (HFD) feeding in mice. Chemical ablation of sympathetic nerves significantly reversed steatosis in just 3 days with improvement in hepatomegaly and hepatic triglyceride content without affecting food intake, energy expenditure or body mass. Whole body chemical sympathectomy lowered hepatic expression of gluconeogenic enzymes and peroxisome proliferator activated receptor (PPAR) alpha (40). PPARs such as PPARα and PPARγ are transcription factors that are central in the regulation of lipid, glucose and amino acid metabolism, maintaining the homeostasis of adipose tissue and hepatocytes (41, 42). Selective hepatic sympathetic denervation using phenol also resulted in similar impact on hepatic gluconeogenic enzymes and led to almost complete resolution of hepatic steatosis in 1 week (40). Mechanistically, hepatic denervation attenuated HFD-increased hepatic expression of *CD36* (a fatty acid transporter), *PPAR*α, and *Diacyl-glycerol O-acyltransferase (DGAT) 1* and *DGAT2*, which encode critical enzymes catalyzing the final step of triglyceride synthesis. Hepatic denervation however did not affect mitochondrial or peroxiosomal β-oxidation of fatty acids (40). Hepatic β-adrenergic activity increases with aging in murine models (39), and aging itself is linked with development of NASH (43). Ghosh et al. reported that older rats accumulated even more fat in livers as compared to younger rats with pharmacologic β-adrenergic stimulation (39). Age-related increase in sympathetic activity may thus be a critical link toward development of NASH. In summary, HFD and aging increase hepatic sympathetic activity elevating hepatic lipid burden, whereas surgical or pharmacological ablation of hepatic sympathetic supply ameliorates hepatic steatosis.

### Sympathetic Nervous System and Hepatic Fibrosis

SNS is overly activated in humans with advanced fibrosis or cirrhosis and in animal models of liver fibrosis. Patients with cirrhosis elicit increased levels of catecholamines in blood and enhanced sympathetic nerve activity (44, 45). In mice, over-activity of SNS worsens CCl_4_-mediated fibrosis, whereas dampened sympathetic tone renders lowered susceptibility (46). Norepinephrine, a type of catecholamine, increases the proliferation of cultured HSCs, which is inhibited by alpha- and beta-adrenergic receptor antagonists. Moreover, murine HSCs produce catecholamine and norepinephrine (12). Norepinephrine promotes HSC proliferation likely through the activation of PI3K and MAPK/ERK signaling cascades. Norepinephrine also induces HSC expression of collagen (12, 47). Oben et al. further showed that treatment with norepinephrine induces proliferation of HSCs *in vivo* in *ob/ob* mice. This catecholamine stimulation also results in increased expression of transforming growth factor beta (TGF-β) along with accentuated fibrosis (12). The above stimulatory effects of catecholamines on HSC proliferation as well as the expression of collagen and TGF-β were also found in cultured human HSCs as reported by Sigala et al. (48). Thus, the SNS branch of ANS is pro-fibrogenic in nature. These findings implicate that pharmacological or surgical disruption of sympathetic supply may be effective in preventing the progression to steatohepatitis and fibrosis.

### Parasympathetic Nervous System and Non-alcoholic Steatohepatitis

Anti-inflammatory effects of the PSNS was first demonstrated in a landmark paper by Borovikova et al. They identified that nicotine, acetylcholine and cholinergic agonist carbachol inhibit LPS-mediated production of tumor necrosis factor (TNF) by peripheral blood macrophages. In addition, acetylcholine can inhibit the release of several other pro-inflammatory cytokines including IL-1β, IL-6, and IL-18, but not anti-inflammatory cytokine IL-10. Their work further revealed that electrical stimulation of vagus nerve decreases LPS-mediated hepatic TNF production whereas vagotomy stimulates TNF production (49). This inhibitory effect of vagal electrical stimulation or acetylcholine receptor (AChR) agonism on hepatic TNF production was validated in ischemia/reperfusion injury model. Inhibition of TNF production was at least in part mediated by decreased nuclear translocation of NF-κB (13). Li et al. reported that vagus nerve flow suppresses LPS/galactosamine-induced hepatocyte injury by attenuation of Kupffer cell activation through the activation of nicotinic α7nAchR receptors (50). PSNS also elicits anti-inflammatory effects in *db/db* and HFD-fed obese mice. Low dose nicotine improved insulin resistance in these mice whereas knockout of α7nAChR exacerbated insulin resistance with pro-inflammatory M1 macrophages infiltrating white adipose tissue (WAT). Nicotine also attenuated the production of pro-inflammatory cytokines in peritoneal macrophages induced by stearic acid or TNF (51).

Direct evidence of the role of parasympathetic anti-inflammatory pathway in NAFLD comes from work done by Nishio et al. They demonstrated that hepatic vagotomy worsened methionine-choline deficient diet induced steatohepatitis. Moreover, vagotomy led to activation of Kupffer cells along with downregulation of hepatic PPARα and an aggravated hepatic pro-inflammatory cytokine profile. Beneficial effect of cholinergic stimulation was mediated by activation of STAT3 pathway and inhibition of nuclear translocation of NF-κB. Chimeric mice with α7nAChR deficiency in Kupffer cells had significant aggravation of steatohepatitis. These mice had increased expression levels of enzymes responsible for fatty acid synthesis such as fatty acid synthase (FASN) (52). Galantamine is a cholinergic activator with a unique dual-mode of action via inhibition of acetylcholinesterase and by modulation of nicotinic acetylcholine receptors, and has been used in patients with Alzheimer's disease (53). Galantamine improves insulin resistance and serum lipid profile in streptozotocin-induced diabetic rats (54). HFD-fed mice treated by galantamine show decreased bodyweight and expression of pro-inflammatory cytokines as well as improved insulin resistance. These changes are accompanied by significant reduction in hepatic triglyceride content and marked improvement in hepatic steatosis and inflammation (55).

Taken together, PSNS plays anti-inflammatory role in multiple sites of the body including the liver primarily through modulation of cytokine production in macrophages and via reduction in hepatic lipid content.

### Parasympathetic Nervous System and Hepatic Fibrosis

The role of PSNS in modulation of hepatic fibrosis remains mostly unclear due to limited number of studies. Given the anti-inflammatory role of PSNS in the setting of NAFLD, it is postulated that PSNS is anti-fibrotic indirectly by inhibition of HSC activation. However, Oben et al. showed that PSNS exerts pro-fibrotic effects. Treatment of HSCs in culture with acetylcholine increases the proliferation and collagen expression of HSCs whereas mecamylamine, an inhibitor of nAChRs receptor, abolishes this response (33). Luo et al. demonstrated that treatment with anisodamine, a non-specific cholinergic inhibitor, improves hepatic inflammation, accompanied by decreased production of TGF-β1, malondialdehyde, and hydroxyproline in the liver of CCl_4_-treated mice (56). Of note, anisodamine is also an anti-oxidant agent (57). Thus, it is unclear whether anisodamine's effects attribute to decreased activity of the PSNS or inhibition of oxidative stress. Further studies are warranted toward a better understanding of the role of PSNS in the pathogenesis and progression of hepatic fibrosis.

## Neurotrophic Factors

Neurotrophic factors are small proteins or polypeptides that play crucial roles in the development, differentiation, migration, and survival of various neurons in central and peripheral nervous system (17, 58, 59). Major families of neurotrophic factors include neurotrophins or nerve growth factors (NGF), ciliary neurotrophic family (CNTF), glial cell line-derived neurotrophic factor (GDNF) family, and neuropoietic cytokines (17, 60). Early studies demonstrated the important role of neurotrophic factors in neuronal regeneration in neurodegenerative diseases (59, 60). Recent evidence has identified extra-neuronal role of neurotrophic factors in many other tissues, such as the liver, pancreas, heart, breast, lung, testis, ovary, etc. (61). Neurotrophic factors also regulate systemic energy homeostasis and insulin sensitivity. GDNF, brain-derived neurotrophic factor (BDNF, a NGF family member), CNTF and Neuregulin-4 (NRG4) are among the most studied neurotrophic factors in the context of NAFLD, and their roles are discussed below.

### Glial Cell-Line Derived Neurotrophic Factor

GDNF is the most studied neurotrophic factor in GDNF family of proteins. Other members include neurturin, artemin and persephin (62). GDNF is a glycosylated, disulfide-bonded homodimer that has distant relation with TGF-β superfamily (63). It binds its cognate GDNF family receptor α_1_ (GFRα1) (64, 65) stimulating intracellular PI3K/AKT and Ras/MAPK pathways (66). GDNF promotes the survival and differentiation of dopaminergic neurons (59, 63). GDNF also acts on non-neuronal cells by regulating spermatogenesis, salivary stem cell survival, and ureteral budding in embryonic kidney (67, 68). Our work has shown that GDNF and its cognate receptor are expressed in white adipose tissue (WAT), brown adipose tissue and the liver, playing an important role in lipid metabolism and the progression of NAFLD (69).

#### Glial Cell-Line Derived Neurotrophic Factor and Non-alcoholic Steatohepatitis

Hepatic deposition of fat leads to an increase in hepatic infiltration by inflammatory cells along with increased synthesis of cytokines such as TNF, IL-1β, and IL-6 (70–72). Thus, tight regulation of lipid metabolism in the liver has an important role in the progression of NAFLD. We used a transgenic mouse strain that overexpresses GDNF in central nervous system, enteric nervous system, WAT, brown adipose tissue and liver as described earlier. We have shown that GDNF has a protective effect on HFD induced obesity and hepatic steatosis (69). *GDNF* transgenic mice are resistant to HFD-induced obesity as well as insulin and leptin resistance. *GDNF* transgenic mice are also protected from developing hepatic steatosis as manifested by decreased steatosis and substantially lower triglyceride accumulation in the liver as compared to controls (20, 69). Mechanistically, overexpression of GDNF in transgenic mice reduced the expression of master regulators of lipid homeostasis, including sterol regulatory element binding transcription factor 1 (*SREBF1*), *PPAR*α*, PPAR*γ, and carnitine palmitoyltransferase 1 (*CPT1*), all of which are elevated in models of NAFLD. Specific analysis of *PPAR*γ promoter has revealed an inhibitory effect of GDNF on *PPAR*γ promoter activity in HepG2 cells (20). The *CD 36* gene, which encodes fatty acid translocase mediating fatty acid uptake in hepatocytes, is under direct control by transcription factor PPARα and PPARγ (42, 73). Transgenic expression of *GDNF* abrogated HFD-induced expression of CD 36 protein. *De novo* lipogenesis machinery was also diminished in *GDNF* transgenic mice as seen by significant reduction in the mRNA levels of *FASN*, stearoyl-CoA desaturase (*SCD-1*) and *DGAT2* while on HFD (20). The protective role of transgenic expression of GDNF against hepatic steatosis was recapitulated in a more therapeutic approach by using GDNF-loaded nanoparticles. Direct role of GDNF in improving hepatic lipid homeostasis has also been confirmed in heterozygous GFRα1 receptor-knockout mice, whereby the expression of *PPAR*α, *PPAR*γ*, CD36, FASN, DGAT2*, and *SCD-1* are all increased (20). Autophagy maintains insulin sensitivity, regulates lipid stores, and protects against the development of inflammation (4). Work in our lab has demonstrated a stimulatory effect of GDNF on hepatic macroautophagy (20, 74). As compared to HFD-fed WT mice, *GDNF* transgenic mice displayed reduced expression of autophagic marker p62/sequestosome-1 and increased expression of autophagy related 5 (Atg5), Beclin 1 and microtubule-associated protein-1 light chain 3 (20). This *in vivo* effect of GDNF was further confirmed by *in vitro* analysis of autophagic flux in primary mouse hepatocytes and secondary rat cell line (74).

In line with reduced hepatic steatosis, *GDNF* transgenic mice resist the development of hepatic inflammation when challenged with HFD (20, 69). GDNF-loaded nanoparticles also successfully attenuated hepatic inflammation in HFD-fed mice (20). Overall, these findings have revealed that GDNF plays a crucial role in HFD-induced hepatic steatosis through its effect on autophagy and critical transcription factors such as PPARα, PPARγ, and Srebf1. Thus, GDNF may be a therapeutic agent in reversing steatosis and steatohepatitis (20, 69). It however remains unexplored as to the source of GDNF in native hepatic tissues. Regardless, it is possible that GDNF functions as a trophic factor for hepatic neurons as well as executing direct effects on hepatic parenchymal and possibly non-parenchymal cells. Tao et al. analyzed human liver tissue specimens and primary cell lines from human livers, and showed that GDNF is likely present in human HSCs as it is colocalized with α-smooth muscle actin positive cells (21).

#### Glial Cell-Line Derived Neurotrophic Factor and Hepatic Fibrosis

Hepatic *GDNF* expression is elevated in patients with advanced liver fibrosis in conditions including NASH, alcoholic liver disease or hepatitis B virus infection. Serum GDNF level is increased in a stepwise fashion with the advancement of fibrosis stages. Similar results were seen in mouse fibrosis models. *GDNF* expression in the liver was increased by 2–4-folds in mice which received bile duct ligation or were fed with methionine-choline deficient diet, and by almost 13-fold in mice treated with CCl_4_ (21). Whether the increase in GDNF plays a protective or exacerbating role in the pathogenesis of hepatic fibrosis remains elusive, and results are conflicting. Work from our laboratory has shown that GDNF negates hepatic fibrogenesis in HFD-fed mice. HFD-fed mice with administration of GDNF-loaded nanoparticles exhibited 4.5-fold less fibrosis as compared to control nanoparticles-treated mice (20). It is unclear if this modulation of hepatic fibrosis by GDNF was due to decreased hepatocyte injury or direct inhibition of HSC activation. Conversely, in the study by Tao et al. mice treated with GDNF-expressing adenovirus showed worsened fibrosis and increased hepatic expression of pro-inflammatory and pro-fibrotic genes in bile duct ligation and CCl_4_ fibrotic models; whereas, GDNF knockdown lowered fibrosis burden. The same study showed that GDNF activated mouse HSCs *in vitro*. This pro-fibrotic role of GDNF in CCl_4_ and bile duct ligation models of hepatic fibrosis is opposite to what was seen in our study with HFD-fed model of NAFLD (20). Although Tao et al. showed increased expression of hepatic *GDNF* in human and animal models of NASH, they did not determine the role of GDNF in the progression or prevention of NASH associated fibrosis. Further work is warranted to clarify the precise role of GDNF in the pathogenesis of liver fibrosis associated with NASH.

### Brain-Derived Neurotrophic Factor

BDNF, a member of neurotrophin family, is the most studied neurotrophic factor due to its abundance in nervous system and brain. BDNF plays important roles in development, differentiation, maintenance, and regeneration of neurons (75, 76). It signals through p75 neurotrophin receptor and tropomyosin receptor kinases (77), which are expressed primarily in neurons as well as in a variety of tissues including the liver, pancreas, colon, breast, immune system, muscles, and prostrate. Multiple studies have shown that BDNF regulates energy homeostasis and the development of NAFLD. BDNF improves blood glucose and insulin resistance as well as hepatomegaly and inflammation in *db/db* mice (78–80). Tsuchida et al. showed that subcutaneous administration of BDNF to *db/db* mice decreased dyslipidemia, hepatic triglyceride content and hepatic steatosis (81). Comparison of BDNF with thiazolidinediones in *db/db* mice revealed that BDNF was superior to thiazolidinediones in decreasing food intake, reducing body weight, and ameliorating hepatic steatosis and hepatomegaly (82). These findings point toward the role of BDNF in improving dysregulated energy homeostasis, insulin resistance and hepatic steatosis. Thus, BDNF could potentially be used as an agent to treat hepatic steatosis. Further studies are needed to establish the beneficial effects of BDNF in the treatment of hepatic steatosis in other models of hepatic steatosis than *db/db* mice.

### Ciliary Neurotrophic Factor

CNTF is a well-studied neurotrophic factor that is structurally related to IL-6 and is highly expressed in central and peripheral nervous system including PSNS. Its attachment to cognate CNTFα receptor initiates heterodimerization of leukemia inhibitory factor and gp130 receptors (83). This heterodimer then transduces signals intracellularly though JAK/STAT or Ras/MAPK pathways (84). CNTF also signals through IL-6 receptor, at least in humans (85). CNTF prevents degeneration and cell death of neuroglia, sensory, motor, hippocampal and cerebellar neurons (83). Nonogaki et al. showed that treatment of rats with CNTF promoted lipolysis and lipid export from liver (86). Sleeman et al. demonstrated that CNTF treatment increased basal metabolic rate of HFD-fed *db/db* mice. CNTF improved hepatomegaly, hepatic steatosis, and inflammation. These effects were associated with decreased hepatic expression of *SCD-1* and *glycerol-3-phosphate acyltransferase 1* that mediate *de novo* lipogenesis as well as increased expression of *PPAR*α and *CPT-1* that promote mitochondrial β-oxidation (87). Cui et al. studied effects of recombinant human CNTF in HFD-fed rat model of obesity and hepatic steatosis. Treatment with CNTF significantly improved hepatic steatosis and serum markers of hepatic inflammation. In *in vitro* cultured HepG2 cells, CNTF increased the levels of *PPAR*α and *CPT-1* and reduced the expression of *FASN, SCD-1*, and *SREBF1*. Overall these changes elucidated a peripheral role of CNTF in decreasing *de novo* lipogenesis and enhancing lipolysis in hepatocytes (88). Despite somewhat discouraging clinical outcomes of CNTF in the treatment of obesity due to various adverse effects and lack of long term effect, it remains an exciting and novel therapeutic agent to modulate hepatic steatosis (84). Modifications of CNTF molecule to improve its pharmacokinetics, efficacy, and reduction of immunogenicity could reignite its role in clinical applications.

### Neuregulin-4

NRG4 is a neurotrophic factor that belongs to Neuregulin family of epidermal growth factor-like ligands and signals through tyrosinase kinase receptor members of the ErbB family (89). It regulates neuron development, adipocyte differentiation, brown adipose tissue thermogenesis, and energy homeostasis (90–92). It is also produced in both brown and white adipose tissues and then transduces signal to hepatocytes in an endocrine fashion through ErbB4 receptors. Expression of NRG4 is significantly higher in brown adipose tissues and expression of NRG4 in adipose tissues is inversely related with obesity in mice and human adipose tissue (92, 93). Decreased production of NRG4 in obesity is likely due to direct impact of pro-inflammatory cytokines produced in obesity such as TNF-α and IL-1β (92, 94). Wang et al. showed that HFD-fed *NRG4* knockout mice had increased adiposity, hypertriglyceridemia and increased insulin resistance (92). These mice also manifested more pronounced hepatic steatosis and increased serum markers of hepatic inflammation as compared to WT mice. Knockout mice had increased hepatic expression of genes associated with *de novo* lipogenesis and inflammation along with increased *SREBF1* expression, whereas the expression of genes associated with beta oxidation of fatty acids and gluconeogenesis was unchanged (92, 95). NRG4 attenuated lipogenesis in cultured hepatocytes *in vitro*. On the other hand, transgenic expression of NRG4 in adipose tissues rendered mice resistant to HFD-induced weight gain as compared to WT mice. These mice also had significantly less hepatic steatosis, inflammation and fibrosis (92, 95), along with increased hepatic beta oxidation of fatty acids and increased ketogenesis (94). Moreover, NRG4 directly affects hepatocyte cell death *in vivo* through downregulation of proteasomal degradation of c-FLIP, a major anti-apoptotic/necrotic protein. *NRG4* knockout mice had decreased hepatic c-FLIP levels whereas *NRG4* transgenic mice had elevated c-FLIP levels. Restoration of hepatic c-FLIP expression in HFD-fed *NRG4* knockout mice using adenovirus blocked the progression of hepatic steatosis to NASH (95). In multiple cross-sectional studies of adults and children with NAFLD, serum NRG4 levels were significantly lower when compared to healthy controls, suggesting an important role of NRG4 in human NALFD as well (96–98). Collectively, these studies demonstrate that adipose tissue-derived NRG4, via an endocrine fashion, can improve hepatic steatosis, inflammation and fibrosis.

## Conclusions

**Figure 1:**
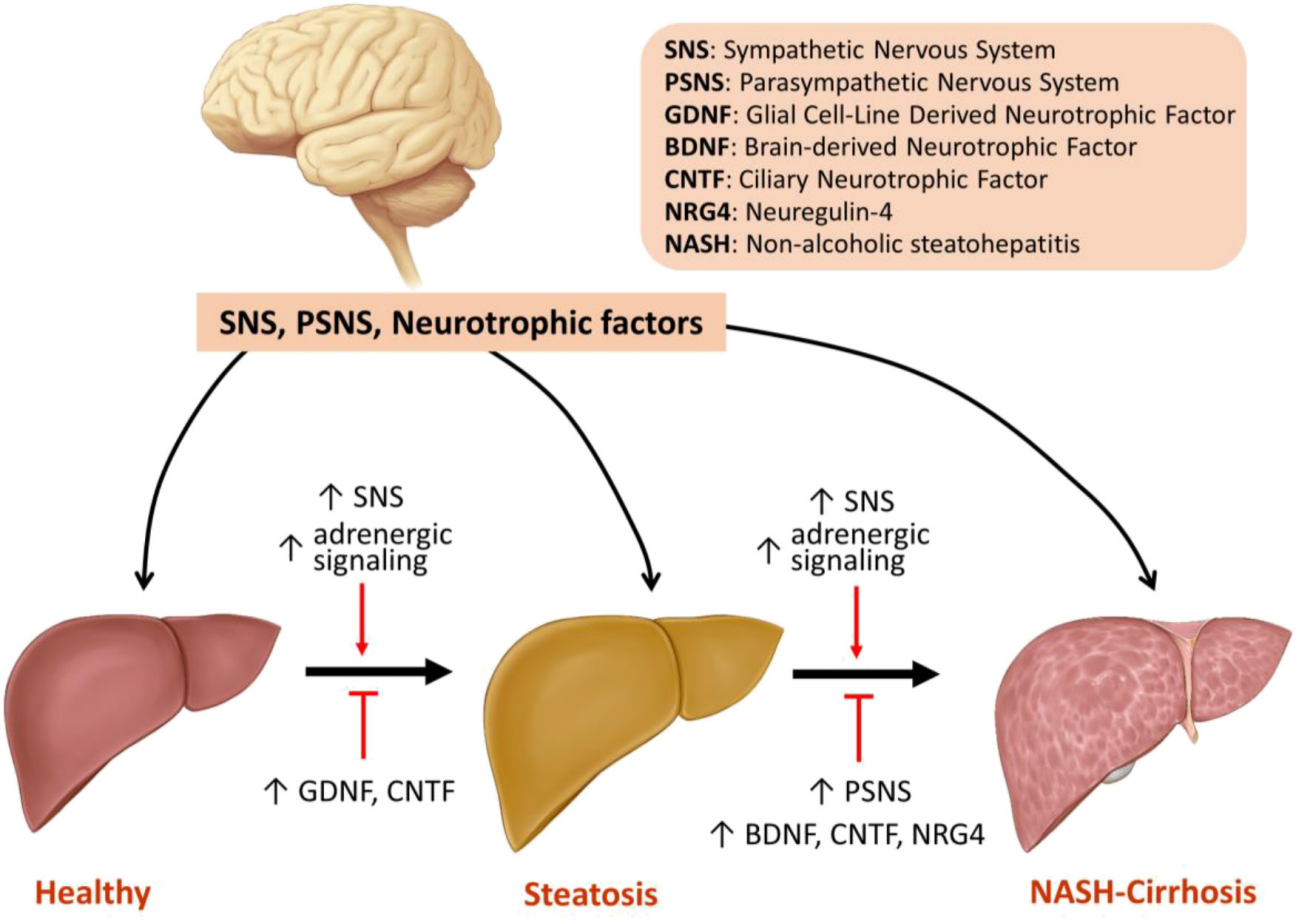
Model of the regulation of steatosis, NASH, and fibrosis by SNS, PSNS and neurotrophic factors.

In conclusion, changes in SNS and PSNS activity as well as the expression of neurotrophic factors play significant roles in the pathogenesis of NAFLD and its progression to NASH and cirrhosis. Activation of SNS and adrenergic signaling promotes steatosis and hepatic fibrosis, whereas stimulation of PSNS improves NASH with its effects on hepatic fibrosis remaining unclear. Increased expression of neurotrophic factors including GDNF, CNTF, BDNF, and NRG4 all alleviate hepatic steatosis. BDNF, CNTF, and NRG4 decrease inflammation, but only NRG4 improves fibrosis (Figure 1, Table 1). The following is a point-by-point summary of the roles of SNS, PSNS, and neurotrophic factors.

- Modulation of the hepatic nervous system and neurotrophic factors play significant roles in management of hepatic steatosis, NASH and hepatic fibrosis.
- SNS is over-activated in the setting of aging, HFD-induced steatosis and advanced hepatic fibrosis.
- SNS stimulation promotes hepatic steatosis whereas surgical or chemical inhibition of SNS improves steatosis.
- SNS activation promotes hepatic fibrosis by enhancing proliferation and activation of HSCs along with increased signaling of TGF-β.
- PSNS activation improves NASH by decreasing hepatic lipid content and by reducing proinflammatory cytokine production in liver. This can be achieved by vagus stimulation or chemical agonism of PSNS.
- Role of PSNS in modulation of hepatic fibrosis remains unclear, with limited data indicating a promoting effect.
- GDNF, CNTF, BDNF, and NRG4 improve insulin resistance and alleviate hepatic steatosis by reducing hepatic lipogenesis and improving β-oxidation.
- GDNF's effects on liver fibrosis are contradictory by playing antifibrotic and profibrotic roles in different models. NRG4 has been shown to ameliorate fibrosis. The role of CNTF and BDNF in hepatic fibrosis is not understood.
- Further studies are needed to better understand the roles of neurotrophic factors and the hepatic nervous system in modulation of NAFLD.

**Table 1:** Key References.

	**Conclusions**	**Models**	**References**
Sympathetic nervous system	Pharmacological sympathetic agonism induces hepatic fibrosis by promoting HSC proliferation	Mouse model of obesity	Oben et al. (12)
	Pharmacological β-adrenergic agonism promotes steatosis	Mouse and rat model of NAFLD	Ghosh et al. (39)
	HFD increases sympathetic activity, and surgical or chemical ablation of hepatic sympathetic supply improves steatosis	Mouse model of NAFLD	Hurr et al. (40)
Parasympathetic nervous system	Pharmacological or electrical parasympathetic agonism dampens proinflammatory cytokine production	Rat model of endotoxemia	Borovikova et al. (49)
	Vagotomy exacerbates steatosis and hepatic inflammation	Mouse model of NASH	Nishio et al. (52)
Neurotrophic factors	GDNF overexpression renders resistance to development of obesity and steatosis	Mouse model of obesity	Mwangi et al. (69)
	BDNF improves steatohepatitis	Mouse model of diabetes	Tonra JR et al. (79)
	CNTF improves hepatic steatosis and inflammation	Mouse model of diabetes	Sleeman et al. (87)
	NRG4 prevents development of steatohepatitis and fibrosis	Mouse model of NASH	Guo et al. (95)

## Author Contributions

MA wrote most of the paper and organized final draft. MY wrote the initial section of the paper. MA, PH, and SS edited and revised the paper.

### Conflict of Interest

The authors declare that the research was conducted in the absence of any commercial or financial relationships that could be construed as a potential conflict of interest.

## References

[1] Estes C Razavi H Loomba R Younossi Z Sanyal AJ. Modeling the epidemic of nonalcoholic fatty liver disease demonstrates an exponential increase in burden of disease. Hepatology. (2018) 67:123–33. 10.1002/hep.29466 28802062 PMC5767767

[2] Arab JP Arrese M Trauner M. Recent insights into the pathogenesis of nonalcoholic fatty liver disease. Annu Rev Pathol. (2018) 13:321–50. 10.1146/annurev-pathol-020117-043617 29414249

[3] Benedict M Zhang X. Non-alcoholic fatty liver disease: an expanded review. World J Hepatol. (2017) 9:715–32. 10.4254/wjh.v9.i16.715 28652891 PMC5468341

[4] Amir M Czaja MJ. Autophagy in nonalcoholic steatohepatitis. Expert Rev Gastroenterol Hepatol. (2011) 5:159–66. 10.1586/egh.11.4 21476911 PMC3104297

[5] Manne V Handa P Kowdley KV. Pathophysiology of nonalcoholic fatty liver disease/nonalcoholic steatohepatitis. Clin Liver Dis. (2018) 22:23–37. 10.1016/j.cld.2017.08.007 29128059

[6] Yin C Evason KJ Asahina K Stainier DY. Hepatic stellate cells in liver development, regeneration, and cancer. J Clin Invest. (2013) 123:1902–10. 10.1172/JCI66369 23635788 PMC3635734

[7] Friedman SL. Mechanisms of hepatic fibrogenesis. Gastroenterology. (2008) 134:1655–69. 10.1053/j.gastro.2008.03.003 18471545 PMC2888539

[8] Taher J Farr S Adeli K. Central nervous system regulation of hepatic lipid and lipoprotein metabolism. Curr Opin Lipidol. (2017) 28:32–8. 10.1097/MOL.0000000000000373 27906714

[9] Jensen KJ Alpini G Glaser S. Hepatic nervous system and neurobiology of the liver. Compr Physiol. (2013) 3:655–65. 10.1002/cphy.c120018 23720325 PMC3733049

[10] Das UN. Obesity: genes, brain, gut, and environment. Nutrition. (2010) 26:459–73. 10.1016/j.nut.2009.09.020 20022465

[11] Exton JH. Mechanisms involved in alpha-adrenergic phenomena: role of calcium ions in actions of catecholamines in liver and other tissues. Am J Physiol. (1980) 238:E3–12. 10.1152/ajpendo.1980.238.1.E3 6243874

[12] Oben JA Roskams T Yang S Lin H Sinelli N Torbenson M. Hepatic fibrogenesis requires sympathetic neurotransmitters. Gut. (2004) 53:438–45. 10.1136/gut.2003.026658 14960531 PMC1773985

[13] Metz CN Pavlov VA. Vagus nerve cholinergic circuitry to the liver and the gastrointestinal tract in the neuroimmune communicatome. Am J Physiol Gastrointest Liver Physiol. (2018) 315:G651–8. 10.1152/ajpgi.00195.2018 30001146 PMC6293249

[14] Kalashnyk OM Gergalova GL Komisarenko SV Skok MV. Intracellular localization of nicotinic acetylcholine receptors in human cell lines. Life Sci. (2012) 91:1033–7. 10.1016/j.lfs.2012.02.005 22365965

[15] Schachtrup C Le Moan N Passino MA Akassoglou K. Hepatic stellate cells and astrocytes: stars of scar formation and tissue repair. Cell Cycle. (2011) 10:1764–71. 10.4161/cc.10.11.15828 21555919 PMC3142460

[16] Mitre M Mariga A Chao MV. Neurotrophin signalling: novel insights into mechanisms and pathophysiology. Clin Sci. (2017) 131:13–23. 10.1042/CS20160044 27908981 PMC5295469

[17] Razavi S Nazem G Mardani M Esfandiari E Salehi H Esfahani SH. Neurotrophic factors and their effects in the treatment of multiple sclerosis. Adv Biomed Res. (2015) 4:53. 10.4103/2277-9175.151570 25802822 PMC4361963

[18] Evans JR Barker RA. Neurotrophic factors as a therapeutic target for Parkinson's disease. Expert Opin Ther Targets. (2008) 12:437–47. 10.1517/14728222.12.4.437 18348680

[19] Cassiman D Denef C Desmet VJ Roskams T. Human and rat hepatic stellate cells express neurotrophins and neurotrophin receptors. Hepatology. (2001) 33:148–58. 10.1053/jhep.2001.20793 11124831

[20] Mwangi SM Peng S Nezami BG Thorn N Farris AB III Jain S. Glial cell line-derived neurotrophic factor protects against high-fat diet-induced hepatic steatosis by suppressing hepatic PPAR-gamma expression. Am J Physiol Gastrointest Liver Physiol. (2016) 310:G103–16. 10.1152/ajpgi.00196.2015 26564715 PMC4719063

[21] Tao L Ma W Wu L Xu M Yang Y Zhang W. Glial cell line-derived neurotrophic factor (GDNF) mediates hepatic stellate cell activation via ALK5/Smad signalling. Gut. (2019) 68:2214–27. 10.1136/gutjnl-2018-317872 31171625 PMC6842044

[22] Ji H Zhang Y Shen XD Gao F Huang CY Abad C. Neuropeptide PACAP in mouse liver ischemia and reperfusion injury: immunomodulation by the cAMP-PKA pathway. Hepatology. (2013) 57:1225–37. 10.1002/hep.25802 22532103 PMC3479352

[23] Li JF Shu JC Tang SH Deng YM Fu MY Lv X. β-Nerve growth factor attenuates hepatocyte injury induced by D-galactosamine *in vitro* via TrkA NGFR. Mol Med Rep. (2013) 8:813–7. 10.3892/mmr.2013.1590 23864198

[24] Kendall TJ Hennedige S Aucott RL Hartland SN Vernon MA Benyon RC. p75 neurotrophin receptor signaling regulates hepatic myofibroblast proliferation and apoptosis in recovery from rodent liver fibrosis. Hepatology. (2009) 49:901–10. 10.1002/hep.22701 19072833

[25] Nakagawa T Tsuchida A Itakura Y Nonomura T Ono M Hirota F. Brain-derived neurotrophic factor regulates glucose metabolism by modulating energy balance in diabetic mice. Diabetes. (2000) 49:436–44. 10.2337/diabetes.49.3.436 10868966

[26] Rezende LF Santos GJ Santos-Silva JC Carneiro EM Boschero AC. Ciliary neurotrophic factor (CNTF) protects non-obese Swiss mice against type 2 diabetes by increasing beta cell mass and reducing insulin clearance. Diabetologia. (2012) 55:1495–504. 10.1007/s00125-012-2493-5 22349107

[27] Fukuda Y Imoto M Koyama Y Miyazawa Y Hayakawa T. Demonstration of noradrenaline-immunoreactive nerve fibres in the liver. J Int Med Res. (1996) 24:466–72. 10.1177/030006059602400603 8959530

[28] Taba Taba Vakili S Kailar R Rahman K Nezami BG Mwangi SM Anania FA Glial cell line-derived neurotrophic factor-induced mice liver defatting: a novel strategy to enable transplantation of steatotic livers. Liver Transpl. (2016) 22:459–67. 10.1002/lt.24385 26714616 PMC4809758

[29] Miyazawa Y Fukuda Y Imoto M Koyama Y Nagura H. Immunohistochemical studies on the distribution of nerve fibers in chronic liver diseases. Am J Gastroenterol. (1988) 83:1108–14. 3048081

[30] Mizuno K Ueno Y. Autonomic nervous system and the liver. Hepatol Res. (2017) 47:160–5. 10.1111/hepr.12760 27272272

[31] Thoene M Rytel L Dzika E Wlodarczyk A Kruminis-Kaszkiel E Konrad P. Bisphenol a Causes liver damage and selectively alters the neurochemical coding of intrahepatic parasympathetic nerves in juvenile porcine models under physiological conditions. Int J Mol Sci. (2017) 18:2726. 10.3390/ijms18122726 29244763 PMC5751327

[32] Akiyoshi H Terada T. Mast cell, myofibroblast and nerve terminal complexes in carbon tetrachloride-induced cirrhotic rat livers. J Hepatol. (1998) 29:112–9. 10.1016/S0168-8278(98)80185-2 9696499

[33] Oben JA Yang S Lin H Ono M Diehl AM. Acetylcholine promotes the proliferation and collagen gene expression of myofibroblastic hepatic stellate cells. Biochem Biophys Res Commun. (2003) 300:172–7. 10.1016/S0006-291X(02)02773-0 12480538

[34] Cardani R Zavanella T. Immunohistochemical localization of beta 1-adrenergic receptors in the liver of male and female F344 rat. Histochem Cell Biol. (2001) 116:441–5. 10.1007/s00418-001-0340-8 11735007

[35] Luo L Xu T Wang P Mao L Xi C Huang J. Expression of muscarinic acetylcholine receptors in hepatocytes from rat fibrotic liver. Exp Toxicol Pathol. (2017) 69:73–81. 10.1016/j.etp.2016.11.005 27899232

[36] Bockx I Vander Elst I Roskams T Cassiman D The hepatic vagus nerve stimulates hepatic stellate cell proliferation in rat acute hepatitis via muscarinic receptor type 2. Liver Int. (2010) 30:693–702. 10.1111/j.1478-3231.2010.02229.x

[37] Li JH Gautam D Han SJ Guettier JM Cui Y Lu H Hepatic muscarinic acetylcholine receptors are not critically involved in maintaining glucose homeostasis in mice. Diabetes. (2009) 58:2776–87. 10.2337/db09-0522 19752163 PMC2780871

[38] Streba LA Vere CC Ionescu AG Streba CT Rogoveanu I. Role of intrahepatic innervation in regulating the activity of liver cells. World J Hepatol. (2014) 6:137–43. 10.4254/wjh.v6.i3.137 24672643 PMC3959114

[39] Ghosh PM Shu ZJ Zhu B Lu Z Ikeno Y Barnes JL. Role of beta-adrenergic receptors in regulation of hepatic fat accumulation during aging. J Endocrinol. (2012) 213:251–61. 10.1530/JOE-11-0406 22457517 PMC3539306

[40] Hurr C Simonyan H Morgan DA Rahmouni K Young CN. Liver sympathetic denervation reverses obesity-induced hepatic steatosis. J Physiol. (2019) 597:4565–80. 10.1113/JP277994 31278754 PMC6716997

[41] Wahli W Braissant O Desvergne B. Peroxisome proliferator activated receptors: transcriptional regulators of adipogenesis, lipid metabolism and more. Chem Biol. (1995) 2:261–6. 10.1016/1074-5521(95)90045-4 9383428

[42] Kersten S. Integrated physiology and systems biology of PPARα. Mol Metab. (2014) 3:354–71. 10.1016/j.molmet.2014.02.002 24944896 PMC4060217

[43] Fontana L Zhao E Amir M Dong H Tanaka K Czaja MJ Aging promotes the development of diet-induced murine steatohepatitis but not steatosis. Hepatology. (2013) 57:995–1004. 10.1002/hep.26099 23081825 PMC3566282

[44] Henriksen JH Moller S Ring-Larsen H Christensen NJ. The sympathetic nervous system in liver disease. J Hepatol. (1998) 29:328–41. 10.1016/S0168-8278(98)80022-6 9722218

[45] Esler M Dudley F Jennings G Debinski H Lambert G Jones P. Increased sympathetic nervous activity and the effects of its inhibition with clonidine in alcoholic cirrhosis. Ann Intern Med. (1992) 116:446–55. 10.7326/0003-4819-116-6-446 1739234

[46] Oben JA Diehl AM. Sympathetic nervous system regulation of liver repair. Anat Rec A Discov Mol Cell Evol Biol. (2004) 280:874–83. 10.1002/ar.a.20081 15382023

[47] Oben JA Yang S Lin H Ono M Diehl AM. Norepinephrine and neuropeptide Y promote proliferation and collagen gene expression of hepatic myofibroblastic stellate cells. Biochem Biophys Res Commun. (2003) 302:685–90. 10.1016/S0006-291X(03)00232-8 12646223

[48] Sigala B McKee C Soeda J Pazienza V Morgan M Lin CI. Sympathetic nervous system catecholamines and neuropeptide Y neurotransmitters are upregulated in human NAFLD and modulate the fibrogenic function of hepatic stellate cells. PLoS ONE. (2013) 8:e72928. 10.1371/journal.pone.0072928 24019886 PMC3760858

[49] Borovikova LV Ivanova S Zhang M Yang H Botchkina GI Watkins LR. Vagus nerve stimulation attenuates the systemic inflammatory response to endotoxin. Nature. (2000) 405:458–62. 10.1038/35013070 10839541

[50] Li Y Xu Z Yu Y Yuan H Xu H Zhu Q. The vagus nerve attenuates fulminant hepatitis by activating the Src kinase in Kuppfer cells. Scand J Immunol. (2014) 79:105–12. 10.1111/sji.12141 24313447

[51] Wang X Yang Z Xue B Shi H. Activation of the cholinergic antiinflammatory pathway ameliorates obesity-induced inflammation and insulin resistance. Endocrinology. (2011) 152:836–46. 10.1210/en.2010-0855 21239433 PMC3040050

[52] Nishio T Taura K Iwaisako K Koyama Y Tanabe K Yamamoto G. Hepatic vagus nerve regulates Kupffer cell activation via alpha7 nicotinic acetylcholine receptor in nonalcoholic steatohepatitis. J Gastroenterol. (2017) 52:965–76. 10.1007/s00535-016-1304-z 28044208

[53] Lilienfeld S. Galantamine–a novel cholinergic drug with a unique dual mode of action for the treatment of patients with Alzheimer's disease. CNS Drug Rev. (2002) 8:159–76. 10.1111/j.1527-3458.2002.tb00221.x 12177686 PMC6741688

[54] Ali MA El-Abhar HS Kamel MA Attia AS. Antidiabetic effect of galantamine: novel effect for a known centrally acting drug. PLoS ONE. (2015) 10:e0134648. 10.1371/journal.pone.0134648 26262991 PMC4532414

[55] Satapathy SK Ochani M Dancho M Hudson LK Rosas-Ballina M Valdes-Ferrer SI. Galantamine alleviates inflammation and other obesity-associated complications in high-fat diet-fed mice. Mol Med. (2011) 17:599–606. 10.2119/molmed.2011.00083 21738953 PMC3146607

[56] Luo L Zhou A. Antifibrotic activity of anisodamine *in vivo* is associated with changed intrahepatic levels of matrix metalloproteinase-2 and its inhibitor tissue inhibitors of metalloproteinases-2 and transforming growth factor beta1 in rats with carbon tetrachloride-induced liver injury. J Gastroenterol Hepatol. (2009) 24:1070–6. 10.1111/j.1440-1746.2008.05756.x 19220677

[57] Poupko JM Baskin SI Moore E. The pharmacological properties of anisodamine. J Appl Toxicol. (2007) 27:116–21. 10.1002/jat.1154 17186568

[58] Gordon T. The role of neurotrophic factors in nerve regeneration. Neurosurg Focus. (2009) 26:E3. 10.3171/FOC.2009.26.2.E3 19228105

[59] Xiao N Le QT. Neurotrophic factors and their potential applications in tissue regeneration. Arch Immunol Ther Exp. (2016) 64:89–99. 10.1007/s00005-015-0376-4 26611762 PMC4805470

[60] Weissmiller AM Wu C. Current advances in using neurotrophic factors to treat neurodegenerative disorders. Transl Neurodegener. (2012) 1:14. 10.1186/2047-9158-1-14 23210531 PMC3542569

[61] Zhao P Qiao J Huang S Zhang Y Liu S Yan LY. Gonadotrophin-induced paracrine regulation of human oocyte maturation by BDNF and GDNF secreted by granulosa cells. Hum Reprod. (2011) 26:695–702. 10.1093/humrep/deq390 21227937

[62] Fielder GC Yang TW Razdan M Li Y Lu J Perry JK. The GDNF family: a role in cancer? Neoplasia. (2018) 20:99–117. 10.1016/j.neo.2017.10.010 29245123 PMC5730419

[63] Lin LF Doherty DH Lile JD Bektesh S Collins F. GDNF: a glial cell line-derived neurotrophic factor for midbrain dopaminergic neurons. Science. (1993) 260:1130–2. 10.1126/science.8493557 8493557

[64] Airaksinen MS Saarma M. The GDNF family: signalling, biological functions and therapeutic value. Nat Rev Neurosci. (2002) 3:383–94. 10.1038/nrn812 11988777

[65] Uesaka T Jain S Yonemura S Uchiyama Y Milbrandt J Enomoto H. Conditional ablation of GFRalpha1 in postmigratory enteric neurons triggers unconventional neuronal death in the colon and causes a Hirschsprung's disease phenotype. Development. (2007) 134:2171–81. 10.1242/dev.001388 17507417

[66] Takahashi M. The GDNF/RET signaling pathway and human diseases. Cytokine Growth Fact Rev. (2001) 12:361–73. 10.1016/S1359-6101(01)00012-0 11544105

[67] Meng X Lindahl M Hyvonen ME Parvinen M de Rooij DG Hess MW. Regulation of cell fate decision of undifferentiated spermatogonia by GDNF. Science. (2000) 287:1489–93. 10.1126/science.287.5457.1489 10688798

[68] Xiao N Lin Y Cao H Sirjani D Giaccia AJ Koong AC. Neurotrophic factor GDNF promotes survival of salivary stem cells. J Clin Invest. (2014) 124:3364–77. 10.1172/JCI74096 25036711 PMC4109543

[69] Mwangi SM Nezami BG Obukwelu B Anitha M Marri S Fu P. Glial cell line-derived neurotrophic factor protects against high-fat diet-induced obesity. Am J Physiol Gastrointest Liver Physiol. (2014) 306:G515–25. 10.1152/ajpgi.00364.2013 24458024 PMC3949027

[70] Shoelson SE Herrero L Naaz A. Obesity, inflammation, and insulin resistance. Gastroenterology. (2007) 132:2169–80. 10.1053/j.gastro.2007.03.059 17498510

[71] Siersbaek R Nielsen R Mandrup S. PPARgamma in adipocyte differentiation and metabolism–novel insights from genome-wide studies. FEBS Lett. (2010) 584:3242–9. 10.1016/j.febslet.2010.06.010 20542036

[72] Pan H Guo J Su Z. Advances in understanding the interrelations between leptin resistance and obesity. Physiol Behav. (2014) 130:157–69. 10.1016/j.physbeh.2014.04.003 24726399

[73] Motojima K Passilly P Peters JM Gonzalez FJ Latruffe N. Expression of putative fatty acid transporter genes are regulated by peroxisome proliferator-activated receptor alpha and gamma activators in a tissue- and inducer-specific manner. J Biol Chem. (1998) 273:16710–4. 10.1074/jbc.273.27.16710 9642225

[74] Mwangi SM Li G Ye L Liu Y Reichardt F Yeligar SM. Glial cell line-derived neurotrophic factor enhances autophagic flux in mouse and rat hepatocytes and protects against palmitate lipotoxicity. Hepatology. (2019) 69:2455–70. 10.1002/hep.30541 30715741 PMC6541506

[75] Binder DK Scharfman HE. Brain-derived neurotrophic factor. Growth Fact. (2004) 22:123–31. 10.1080/08977190410001723308 15518235 PMC2504526

[76] McGregor CE English AW. The role of BDNF in peripheral nerve regeneration: activity-dependent treatments and val66Met. Front Cell Neurosci. (2018) 12:522. 10.3389/fncel.2018.00522 30687012 PMC6336700

[77] Tsuchida A Nakagawa T Itakura Y Ichihara J Ogawa W Kasuga M. The effects of brain-derived neurotrophic factor on insulin signal transduction in the liver of diabetic mice. Diabetologia. (2001) 44:555–66. 10.1007/s001250051661 11380073

[78] Ono M Ichihara J Nonomura T Itakura Y Taiji M Nakayama C Brain-derived neurotrophic factor reduces blood glucose level in obese diabetic mice but not in normal mice. Biochem Biophys Res Commun. (1997) 238:633–7. 10.1006/bbrc.1997.7220 9299565

[79] Tonra JR Ono M Liu X Garcia K Jackson C Yancopoulos GD. Brain-derived neurotrophic factor improves blood glucose control and alleviates fasting hyperglycemia in C57BLKS-Lepr(db)/lepr(db) mice. Diabetes. (1999) 48:588–94. 10.2337/diabetes.48.3.588 10078561

[80] Ono M Itakura Y Nonomura T Nakagawa T Nakayama C Taiji M. Intermittent administration of brain-derived neurotrophic factor ameliorates glucose metabolism in obese diabetic mice. Metabolism. (2000) 49:129–33. 10.1016/S0026-0495(00)90988-0 10647076

[81] Tsuchida A Nonomura T Nakagawa T Itakura Y Ono-Kishino M Yamanaka M. Brain-derived neurotrophic factor ameliorates lipid metabolism in diabetic mice. Diabetes Obes Metab. (2002) 4:262–9. 10.1046/j.1463-1326.2002.00206.x 12099975

[82] Yamanaka M Itakura Y Tsuchida A Nakagawa T Noguchi H Taiji M. Comparison of the antidiabetic effects of brain-derived neurotrophic factor and thiazolidinediones in obese diabetic mice. Diabetes Obes Metab. (2007) 9:879–88. 10.1111/j.1463-1326.2006.00675.x 17924870

[83] Richardson PM. Ciliary neurotrophic factor: a review. Pharmacol Ther. (1994) 63:187–98. 10.1016/0163-7258(94)90045-0 7809179

[84] Pasquin S Sharma M Gauchat JF. Ciliary neurotrophic factor (CNTF): new facets of an old molecule for treating neurodegenerative and metabolic syndrome pathologies. Cytokine Growth Fact Rev. (2015) 26:507–15. 10.1016/j.cytogfr.2015.07.007 26187860

[85] Schuster B Kovaleva M Sun Y Regenhard P Matthews V Grotzinger J. Signaling of human ciliary neurotrophic factor (CNTF) revisited. The interleukin-6 receptor can serve as an alpha-receptor for CTNF. J Biol Chem. (2003) 278:9528–35. 10.1074/jbc.M210044200 12643274

[86] Nonogaki K Pan XM Moser AH Shigenaga J Staprans I Sakamoto N. LIF and CNTF, which share the gp130 transduction system, stimulate hepatic lipid metabolism in rats. Am J Physiol. (1996) 271:E521–8. 10.1152/ajpendo.1996.271.3.E521 8843746

[87] Sleeman MW Garcia K Liu R Murray JD Malinova L Moncrieffe M. Ciliary neurotrophic factor improves diabetic parameters and hepatic steatosis and increases basal metabolic rate in db/db mice. Proc Natl Acad Sci USA. (2003) 100:14297–302. 10.1073/pnas.2335926100 14610276 PMC283586

[88] Cui MX Yang LN Wang XX Wang L Li RL Han W. Alleviative effect of ciliary neurotrophic factor analogue on high fat-induced hepatic steatosis is partially independent of the central regulation. Clin Exp Pharmacol Physiol. (2017) 44:395–402. 10.1111/1440-1681.12709 27973757

[89] Britsch S. The neuregulin-I/ErbB signaling system in development and disease. Adv Anat Embryol Cell Biol. (2007) 190:1–65. 10.1007/978-3-540-37107-6_1 17432114

[90] Paramo B Wyatt S Davies AM. An essential role for neuregulin-4 in the growth and elaboration of developing neocortical pyramidal dendrites. Exp Neurol. (2018) 302:85–92. 10.1016/j.expneurol.2018.01.002 29317193 PMC5866123

[91] Paramo B Wyatt S Davies AM Neuregulin-4 is required for the growth and elaboration of striatal medium spiny neuron dendrites. J Neuropathol Exp Neurol. (2019) 78:725–34. 10.1093/jnen/nlz046 PMC6640913 31225596

[92] Wang GX Zhao XY Meng ZX Kern M Dietrich A Chen Z. The brown fat-enriched secreted factor Nrg4 preserves metabolic homeostasis through attenuation of hepatic lipogenesis. Nat Med. (2014) 20:1436–43. 10.1038/nm.3713 25401691 PMC4257907

[93] Khan RS Newsome PN. NAFLD in 2017: novel insights into mechanisms of disease progression. Nat Rev Gastroenterol Hepatol. (2018) 15:71–2. 10.1038/nrgastro.2017.181 29300050

[94] Chen Z Wang GX Ma SL Jung DY Ha H Altamimi T. Nrg4 promotes fuel oxidation and a healthy adipokine profile to ameliorate diet-induced metabolic disorders. Mol Metab. (2017) 6:863–72. 10.1016/j.molmet.2017.03.016 28752050 PMC5518721

[95] Guo L Zhang P Chen Z Xia H Li S Zhang Y. Hepatic neuregulin 4 signaling defines an endocrine checkpoint for steatosis-to-NASH progression. J Clin Invest. (2017) 127:4449–61. 10.1172/JCI96324 29106384 PMC5707158

[96] Cai C Lin M Xu Y Li X Yang S Zhang H. Association of circulating neuregulin 4 with metabolic syndrome in obese adults: a cross-sectional study. BMC Med. (2016) 14:165. 10.1186/s12916-016-0703-6 27772531 PMC5075753

[97] Yan P Xu Y Wan Q Feng J Li H Yang J. Plasma neuregulin 4 levels are associated with metabolic syndrome in patients newly diagnosed with type 2 diabetes mellitus. Dis Markers. (2018) 2018:6974191. 10.1155/2018/6974191 29721105 PMC5867541

[98] Wang R Yang F Qing L Huang R Liu Q Li X. Decreased serum neuregulin 4 levels associated with non-alcoholic fatty liver disease in children with obesity. Clin Obes. (2019) 9:e12289. 10.1111/cob.12289 30411515

